# Low-Level Laser Therapy for Gingival Inflammation in Children and Adolescents: A Narrative Review Based on In Vitro, In Vivo and Clinical Studies

**DOI:** 10.3390/healthcare14111533

**Published:** 2026-06-01

**Authors:** Mădălina-Ștefania Stancu, Mihaela Tănase, Aneta Munteanu, Ion-Victor Feraru, Carmen Nicoleta Novac, Anca Andreea Boboc, Petra Șurlin, Liviu Steier, Andreea Cristiana Didilescu

**Affiliations:** 1 Department of Pedodontics, Faculty of Dentistry, Carol Davila University of Medicine and Pharmacy, 041292 Bucharest, Romania; madalina-stefania.stancu@drd.umfcd.ro (M.-Ș.S.); mihaela.tanase@umfcd.ro (M.T.); aneta.munteanu@umfcd.ro (A.M.); 2 Department of Pediatrics, Faculty of Medicine, Carol Davila University of Medicine and Pharmacy and Marie Curie Emergency Children’s Hospital, 041451 Bucharest, Romania; dr.carmen.novac@gmail.com (C.N.N.); anca.orzan@umfcd.ro (A.A.B.); 3 Department of Periodontology, Research Center of Periodontal-Systemic Interactions, Faculty of Dental Medicine, University of Medicine and Pharmacy of Craiova, 200349 Craiova, Romania; surlinpetra@gmail.com; 4 Department of Preventive and Restorative Sciences, Robert Schattner Center, School of Dental Medicine, University of Pennsylvania, Philadelphia, PA 19104, USA; lsteier@upenn.edu; 5 Department of Embryology and Microbiology, Faculty of Dentistry, Carol Davila University of Medicine and Pharmacy, 050474 Bucharest, Romania; andreea.didilescu@umfcd.ro

**Keywords:** low-level laser therapy, photobiomodulation, gingivitis, gingival inflammation, pediatric dentistry, oral health, children

## Abstract

**Highlights:**

**What are the main findings?**

- Low-level laser therapy (LLLT) shows anti-inflammatory effects and may promote gingival tissue healing in experimental and clinical studies.
- Limited pediatric clinical evidence suggests that adjunctive LLLT may improve gingival indices when combined with conventional therapy; however, standardized safety reporting remains insufficient.

**What are the implications of the main findings?**

- LLLT may represent a promising non-invasive adjunctive approach for managing gingival inflammation in children and adolescents.
- LLLT may contribute to improving oral health outcomes and patient compliance in pediatric healthcare settings.
- Further well-designed pediatric clinical trials with standardized protocols, systematic safety monitoring, and long-term follow-up are required to support routine clinical implementation.

**Abstract:**

**Background/Objectives:** Gingivitis is one of the most prevalent inflammatory conditions in pediatric populations and represents a significant oral health burden worldwide. In addition to conventional approaches, emerging adjunctive therapies such as low-level laser therapy (LLLT), also known as photobiomodulation, have gained increasing attention. This review aims to evaluate current evidence regarding the clinical relevance and potential healthcare implications of LLLT in the management of gingival inflammation in children and adolescents. **Methods:** A structured narrative literature review was conducted, including experimental, animal, and clinical studies published between 2005 and 2025. Study selection and screening were performed independently by two reviewers. Methodological characteristics of the included pediatric clinical studies were qualitatively assessed, focusing on randomization, blinding, follow-up reporting, and adverse event monitoring. **Results:** Experimental studies demonstrate that LLLT enhances gingival cell proliferation and modulates inflammatory responses, while antimicrobial effects remain inconsistent. Animal studies indicate reductions in gingival inflammation and alveolar bone loss when LLLT is used adjunctively with conventional therapy. Clinical studies in pediatric populations report improvements in gingival indices and periodontal treatment needs, with no adverse effects observed. **Conclusions:** LLLT appears to be a safe, non-invasive adjunctive therapy with potential to improve oral health outcomes in pediatric populations. Its clinical applicability and high patient acceptability support its potential integration into broader oral healthcare strategies. However, further well-designed clinical trials with standardized protocols and long-term follow-up are required.

## 1. Introduction

Gingivitis is an inflammatory condition of the gingival tissues primarily caused by the accumulation of bacterial biofilm at the gingival margin [1,2]. This condition is highly prevalent in children, with nearly all children exhibiting clinical signs of gingival inflammation by puberty [3]. Dental plaque is the determining factor in producing gingivitis [2,4,5].

There are also several predisposing factors which are involved in the etiology of gingival inflammation during childhood and adolescence: dietary habits, medications, systemic illnesses (e.g., diabetes mellitus, thyroid disorders, leukemia), the presence of mixed dentition, inadequate and/or unsupervised oral hygiene routines, and misalignment of the teeth [6].

Clinically, gingival tissues with gingivitis are characterized by swelling, redness, tenderness, a shiny surface, and bleeding upon gentle probing [2]. Gingivitis is a painless condition and rarely causes spontaneous bleeding, but gums can bleed during flossing or brushing. As a result, gingival inflammation in children and adolescents often goes unnoticed by the parents [2,7,8]. On the other hand, in some cases normal clinical features can be confused with pathological ones due to the morphological and structural particularities of the healthy gingiva in children (reddish, lack of stippling, with rounded gingival margins) [9].

Gingivitis prevalence varies by age and environment. The condition often begins in childhood, progresses quickly, and tends to become more frequent and more severe with age [6]. Studies show significant variation in gingivitis prevalence among children: from 28.58% in Chinese children aged 6–12 [7] to over 88% in Bulgarian children aged 10–14 [10], and around 91% in Romanian schoolchildren aged 10–17 [11].

Conventional treatment approaches for gingival inflammation in pediatric patients involve the use of self-performed oral hygiene and professional dental cleaning [12]. There are also adjunctive methods that can help reduce gingivitis, including antimicrobial agents [12], plant extracts [13], probiotics [14] that can be used at home and phototherapy with low-level laser therapy (LLLT), which needs professional evaluation [15]. Laser treatment is well-received by children and parents due to its minimally invasive nature. Studies have demonstrated that children are more compliant during restorative, pulpal, and surgical procedures when lasers are used [16,17].

LLLT is generally considered a minimally invasive and well-tolerated therapeutic approach, which may be advantageous in pediatric patients. However, direct evidence regarding treatment acceptability and patient-reported outcomes in children remains limited.

Despite the high prevalence of gingival inflammation in children and adolescents, clinical evidence regarding the use of LLLT for pediatric gingival inflammation remains limited. Most currently available evidence originates from in vitro studies, animal models, or adult periodontal research, while pediatric clinical trials remain scarce and methodologically heterogeneous.

Given the growing interest in LLLT and the heterogeneity of available evidence, a comprehensive synthesis of current data is needed. Therefore, this narrative review aims to evaluate the experimental and clinical evidence regarding the use of LLLT in pediatric gingival inflammation and to discuss its potential clinical applications.

## 2. Materials and Methods

### 2.1. Study Design

This study was conducted as a structured narrative review aiming to provide a comprehensive overview of the current evidence regarding the use of LLLT, also referred to as photobiomodulation therapy (PBMT), in the management of gingival inflammation in children and adolescents.

The review was designed to integrate and critically discuss findings from in vitro studies, animal models, and pediatric clinical investigations in order to provide a translational perspective on the potential applications of LLLT in pediatric dentistry.

A narrative approach was considered appropriate due to the substantial heterogeneity of the available literature regarding study design, experimental models, laser parameters, treatment protocols, and outcome measures, which precluded the performance of a formal systematic review or quantitative meta-analysis.

The review methodology was conducted according to the principles of the Scale for the Assessment of Narrative Review Articles (SANRA), with particular attention to methodological transparency, literature search strategy, study selection, and critical interpretation of the findings.

As this study was conducted as a structured narrative review rather than a systematic review, prospective protocol registration (e.g., PROSPERO) was not performed.

### 2.2. Search Strategy

A structured literature search was conducted in the following electronic databases: PubMed, Scopus, Web of Science, Cochrane Library, and Google Scholar. The search included studies published between January 2005 and December 2025.

The search strategy combined Medical Subject Headings (MeSH) terms and free-text keywords related to LLLT and pediatric gingival inflammation. The main search terms included: “low-level laser therapy”, “photobiomodulation”, “photobiomodulation therapy”, “gingivitis”, “gingival inflammation”, “pediatric dentistry”, “children”, “adolescents”, “in vitro studies”, “animal models”, and “adjunctive therapy”.

Boolean operators (“AND”, “OR”) were used to combine search terms. Examples of search combinations included: (“low-level laser therapy” OR “photobiomodulation”) AND (“gingivitis” OR “gingival inflammation”) AND (“children” OR “adolescents” OR “pediatric dentistry”).

Additional searches were performed using combinations adapted to the indexing system of each database. Furthermore, a manual search (hand-searching) of the reference lists of relevant articles and review papers was conducted to identify additional eligible studies that may not have been retrieved through the electronic database search.

### 2.3. Study Selection

Study selection was performed in two stages. First, the titles and abstracts of the retrieved articles were screened for relevance according to the predefined eligibility criteria. Subsequently, full-text evaluation was conducted for studies considered potentially eligible.

The screening and selection process was performed independently by two reviewers (A.C.D. and M.Ș.S.). Any disagreements regarding study eligibility were resolved through discussion and consensus among the authors.

Studies were included if they investigated the effects of LLLT on gingival inflammation using in vitro models, animal studies, or pediatric clinical studies.

The review question and eligibility criteria were structured according to PICO-related components. The population of interest included children and adolescents, experimental animal models relevant to gingival inflammation or periodontitis, and in vitro cellular or microbial models. Interventions involved LLLT applied alone or as an adjunct to conventional therapy, while comparators included no treatment, sham irradiation, mechanical debridement, scaling and root planing, or alternative adjunctive therapies. Outcomes of interest included clinical inflammatory indices, microbial reduction, histological and cellular changes, tissue healing, and adverse event reporting.

For each eligible study, the following data were extracted and summarized: author, year of publication, study design, sample characteristics, laser parameters, irradiation protocols, evaluated outcomes, and main findings. The included studies were subsequently grouped according to study type (in vitro studies, animal studies, and pediatric clinical studies) in order to facilitate qualitative narrative synthesis and critical interpretation of the available evidence.

**Figure 1:**
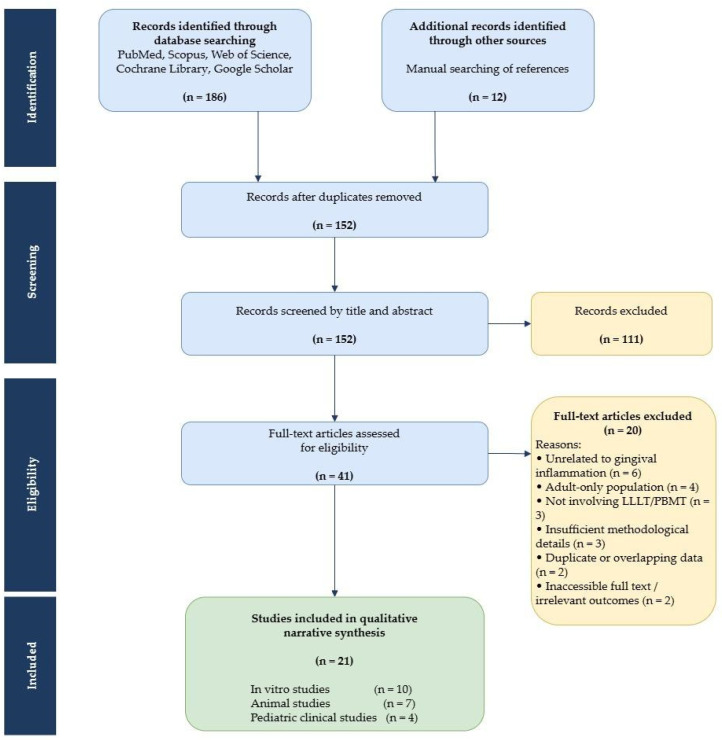
PRISMA-style flow diagram.

The study selection process is summarized in a PRISMA-style flow diagram (Figure 1).

### 2.4. Eligibility Criteria

Studies were selected based on predefined inclusion and exclusion criteria. The inclusion criteria were

- Clinical studies involving children or adolescents, relevant to pediatric gingival inflammation, evaluating the effects of LLLT or PBMT;
- In vitro or in vivo animal studies, testing the effects of LLLT or PBMT on gingival/periodontal tissues;
- Relevant review articles used for contextual discussion;
- Articles published in English.

The exclusion criteria were

- Case reports, conference abstracts, editorials, and letters to the editor;
- Studies lacking sufficient methodological or outcome-related information.

### 2.5. Data Extraction and Synthesis

Relevant studies were initially screened based on title and abstract, followed by full-text evaluation according to the predefined eligibility criteria.

For each included study, the following information was extracted when available: study design, study population or experimental model, laser characteristics (wavelength, power output, energy density, irradiation mode, and exposure duration), intervention protocols, outcome measures, and main findings related to gingival inflammation and tissue response.

Due to the substantial heterogeneity among the included studies regarding experimental design, laser parameters, treatment protocols, outcome assessment methods, and follow-up periods, a qualitative narrative synthesis was considered more appropriate than a quantitative meta-analysis.

The included studies were subsequently grouped into three categories according to the level and type of evidence:

- In vitro studies;
- In vivo animal studies;
- Clinical studies involving pediatric populations.

This approach allowed a structured translational interpretation of the available evidence, from cellular and experimental mechanisms to potential clinical applicability in pediatric dentistry.

### 2.6. Quality Assessment

Given the narrative nature of this review and the substantial heterogeneity of the included studies, a formal standardized risk-of-bias assessment using instruments such as Cochrane RoB, ROBINS-I, or GRADE was not considered appropriate.

Nevertheless, the methodological characteristics of the included pediatric clinical studies were descriptively evaluated, with particular attention to randomization methods, blinding procedures, follow-up reporting, sample size, and adverse event monitoring.

The identified methodological limitations and sources of heterogeneity were further considered during the qualitative interpretation of the findings and are commented on in the Section 4.

## 3. Results

This review comprises 21 studies: 10 in vitro studies, seven in vivo studies on animal models and four randomized clinical trials on pediatric patients.

### 3.1. In Vitro Studies

All 10 in vitro studies investigated the effects of LLLT or PBM on various oral cell types or microbial biofilms (Table 1). The majority of the studies used cultured human gingival fibroblasts (hGFs) [18,19,20,21,22,23,24], while others employed periodontal ligament cells [25], mesenchymal stem cells [26], or human gingival keratinocytes exposed to viable oral microorganisms [27]. In addition, microbial biofilm models were applied either as single- or dual-species systems (Streptococcus mutans and Candida albicans) [28] or as multispecies subgingival biofilms representative of periodontal disease [29].

Laser wavelengths ranged from 615 nm to 1064 nm, with 810 nm being the most frequently studied. Energy densities varied across experiments, typically ranging from 0.5 J/cm^2^ to 20 J/cm^2^, with many studies applying single-dose or daily irradiation protocols for up to three consecutive days. Reported power outputs varied across studies, and both single-wavelength and combined-wavelength approaches were assessed.

### 3.2. In Vivo Studies on Animals

A total of seven in vivo animal studies were included, consisting of two canine models and five rodent models (Table 2). The number of animals used in the experiments ranged from 40 to 180 in rat studies and from 45 to 47 in dog studies. All studies evaluated the effectiveness of low-level laser therapy (LLLT) in the treatment of experimental periodontitis, either as a standalone therapy or as an adjunct to scaling and root planing.

In the rat models, periodontitis was consistently induced using ligatures around the mandibular first molars, followed by various treatment interventions such as SRP, LLLT, and adjunctive therapies like simvastatin or antimicrobial photodynamic therapy (aPDT). One study additionally investigated the effects of dexamethasone-induced immunosuppression [30], and another included in vitro fibroblast assays alongside in vivo assessments [31].

The laser parameters varied across studies on rats, with wavelengths ranging from 660 nm to 808 nm and energy doses from 2 J to 57.14 J/cm^2^. Follow-up durations in these studies varied from 7 to 30 days, with assessments based on clinical, radiographic, histological, and immunological outcomes.

The canine models involved clinical trials simulating veterinary dental prophylaxis procedures. Both studies employed split-mouth designs, applying LLLT to one half of the mouth while the other half served as control. LLLT was applied immediately after dental cleaning, and gingival inflammation was assessed at multiple time points up to 15 days. The laser settings in dogs included 650 nm and 980 nm wavelengths, with energy doses between 6.2 J/cm^2^ and 20 J/cm^2^ [32,33].

**Table 2:** Evaluation of effectiveness of low-level laser therapy in animal models.

Study	Design	Samples	Laser Parameters	Irradiation Protocol	Results	Observations
Alves et al. (2024) [32]	In vivo, randomized, controlled, double-blind experimental study	47 dogs Each dog’s mouth divided into two halves (split-mouth) Control side (left): dental prophylaxis only Treatment side (right): dental prophylaxis + single photobiomodulation session	Source: laser (PBMT) Wavelength: 980 nm Fluence: 6.2 J/cm^2^ Power output: 3.5 W Irradiance: 2.3 W/cm^2^ Exposure duration: 93 s	Canine dental prophylaxis performed on both hemiarches. Single PBMT session applied immediately after prophylaxis on treatment side.	PBMT-treated side showed significantly lower gingivitis scores compared to control from Day 1 through Day 15 (*p* < 0.05). Age and breed influenced PD and calculus scores.	Clinical scores of periodontal diseases (PDs), gingivitis and calculus assessed on Days 1, 3, 8, and 15 post-treatments; blind evaluation. Single PBMT session provided early and sustained reduction in gingival inflammation.
Watson & Brundage (2023) [33]	In vivo, randomized, controlled experimental study	45 dogs (split-mouth) CG (control group): Mock gingival treatment without laser (*n* = 15) LTG (left-treated group): Left dental arcade irradiated (*n* = 15) RTG (right-treated group): Right arcade irradiated (*n* = 15)	Source: GaAlInP laser Wavelength: 650 nm Fluence: 20 J/cm^2^ Power output: 0.1 W Irradiance: 0.2 W/cm^2^ Radiant energy: 10 J per point Exposure duration: 100 s.	Canine dental prophylaxis followed by assessment of gingival inflammation 24 h after treatment. Four irradiation points applied per treated arcade, immediately post-prophylaxis.	LTG showed significant reduction in composite gingival inflammation (*p* = 0.008) and erythema (*p* = 0.030) vs. CG on treated left arcade. Combined LTG + RTG had lower inflammation (*p* = 0.025) and erythema (*p* = 0.013) vs. CG.	Single PBMT session effectively reduced inflammation and redness 24 h after treatment. Supports PBMT as an effective adjunct to dental prophylaxis.
da Cruz Galhardo Camargo et al. (2022) [31]	Combined in vivo and in vitro experimental study	48 rats Control: no periodontitis PDC (periodontitis, no laser) PD + L (periodontitis + infrared laser therapy)	Source: diode laser Wavelength: 790 nm Fluence: 2 J/cm^2^ (in vivo); 4 J/cm^2^ (in vitro) Power output: 100 mW	Ligature-induced periodontitis in rats on mandibular first molars. Infrared light laser therapy (ILLT) applied after 4 weeks. In vitro, NIH/3T3 fibroblasts exposed to 4 J/cm^2^ of infrared laser.	In vivo: PD + L group maintained alveolar bone microstructure compared to PDC on Day 30. In vitro: Laser exposure preserved fibroblast viability and enabled complete wound healing at 4 J/cm^2^.	Outcomes assessed by micro-CT, histology (PMNs) and NIH/3T3 fibroblast viability (in vitro). ILLT effectively preserved bone structure during periodontitis in rats and maintained fibroblast viability and migration in vitro, indicating positive effects on periodontal regeneration.
Pereira et al. (2020) [34]	In vivo, randomized, controlled experimental study	40 Wistar rats Control: no ligature, no treatment Periodontal disease: ligature, no SRP or laser SRP only GL660: SRP + 660 nm diode laser irradiation GL808: SRP + 808 nm diode laser irradiation	Source1: InGaAlP laser Wavelength: 660 nm Source2: GaAlAs laser Wavelength: 808 nm Fluence: 60 J/cm^2^, Power output: 0.03 W Radiant energy: 1.8 J per point Cumulative dose: 10.8 J Exposure duration: 60 s	Periodontitis induced via ligature around mandibular first molar. GL660 group: InGaAlP 660 nm laser irradiation at 6 points immediately after SRP. GL808 group: GaAlAs 808 nm laser irradiation similarly applied.	GL660 group showed significantly less alveolar bone loss compared to PD and SRP alone at both 7 and 14 days. GL660 had higher alveolar bone margin than all other groups on Day 14. GL808 also improved outcomes vs. PD and SRP but was less effective than GL660.	Photobiomodulation with 660 nm laser significantly enhanced periodontal tissue healing and bone preservation post-SRP, demonstrating wavelength-dependent beneficial effect.
Theodoro et al. (2017) [35]	In vivo, randomized, controlled experimental study	150 rats EP (no treatment) 5FU-5-fluorouracil (systemic 5-FU only, 80 + 40 mg/kg) 5FU + SRP 5FU + SRP + LLLT 5FU +SRP + aPDT *n* = 30/each group	Source: InGaAlP laser Wavelength: 660 nm Fluence: 29.4 J/cm^2^ Power output: 0.035 W Irradiance: 1.23 W/cm^2^ Exposure duration: 24 s	aPDT: same laser + methylene blue irrigation after SRP. Experimental periodontitis (EP) induced via ligature around mandibular first molar for 7 days, followed by ligature removal and treatments.	Bone loss: 5FU/SRP/aPDT showed significantly less alveolar bone loss vs. 5FU at 7 days (*p* < 0.05). Lower TNF-α and IL-6 in both LLLT and aPDT groups; increased IL-10 in aPDT group at Day 30.	Both LLLT and aPDT mitigated periodontal damage induced by 5-FU when used after SRP. aPDT was superior in controlling bone loss and modulating inflammatory response, reducing pro-inflammatory cytokines and increasing anti-inflammatory IL-10.
Swerts et al. (2017) [36]	In vivo, randomized, controlled experimental study	180 Wistar rats Vehicle + no treatment Vehicle + SRP Vehicle + SRP + LLLT Simvastatin + no treatment Simvastatin + SRP Simvastatin + SRP + LLLT	Source: GaAlAs laser Wavelength: 660 nm Fluence: 57.14 J/cm^2^ Power output: 0.03 W Irradiance: 0.428 W/cm^2^ Radiant energy: 4 J per point Exposure duration: 133 s	Ligature-induced periodontitis in rats. Simvastatin administered orally at 20 mg/kg daily. SRP performed on ligatured teeth.	LLLT groups showed significantly reduced alveolar bone loss compared to no treatment and SRP alone groups. Simvastatin improved antioxidant markers (increased glutathione, decreased malondialdehyde and carbonylated proteins). Combined simvastatin + LLLT showed enhanced protection against periodontal damage.	LLLT enhances periodontal healing and bone preservation when combined with SRP, and simvastatin modulates oxidative stress favorably; combined therapy offers synergistic benefits for periodontal disease management.
Garcia et al. (2010) [30]	In vivo, randomized, controlled experimental study	120 Wistar rats D-SRP (dexamethasone + SRP + saline) D-SRP + LLLT ND-SRP (no dexamethasone + SRP + saline) ND-SRP + LLLT	Source: GaAlAs laser (LLLT) Wavelength: 660 nm Fluence: 57.14 J/cm^2^ per point Radiant energy: 4 J per point Cumulative dose: 24 J Irradiance: 0.428 W/cm^2^	Ligature-induced periodontitis around mandibular first molars for 7 days, followed by ligature removal, SRP, and subsequent laser therapy or saline irrigation.	SRP + LLLT consistently resulted in significantly lower alveolar bone loss compared to SRP alone in both D and ND groups across all time points (*p* < 0.05 in radiographic and histometric evaluations).	LLLT enhanced bone preservation in both healthy and dexamethasone-compromised rats.

PBMT—photobiomodulation therapy; PD—periodontal disease; CG—control group; LTG—left-treated group; RTG—right-treated group; GaAlInP—gallium–aluminum–indium–phosphide; PDC—periodontitis control (no laser therapy); PD + L—periodontitis plus infrared laser therapy; ILLT—infrared light laser therapy; NIH/3T3—mouse embryonic fibroblast cell line; micro-CT—micro-computed tomography; PMNs—polymorphonuclear neutrophils; SRP—scaling and root planing; GL660—660 nm laser group; GL808—808 nm laser group; InGaAlP—indium–gallium–aluminum–phosphide; GaAlAs—gallium–aluminum–arsenide; EP—experimental periodontitis; 5FU—5-fluorouracil; LLLT—low-level laser therapy; aPDT—antimicrobial photodynamic therapy; TNF-α—tumor necrosis factor-alpha; IL-6—interleukin-6; IL-10—interleukin-10; D—dexamethasone; ND—no dexamethasone.

### 3.3. Clinical Studies on Children and Adolescents

A total of four human clinical studies were included in this section evaluating the effectiveness of LLLT in children and adolescents with gingivitis (Table 3). The age of participants varied between 7 and 19 years. The number of participants per study varied from 13 to 130 individuals, and gingivitis was either chronic catarrhal [37] or associated with fixed orthodontic appliances [38,39].

Two of the studies on children investigated LLLT as an adjunct to basic oral hygiene and plaque removal [37,40], one compared LLLT with topical hyaluronic acid gel [37], and another study evaluated aPDT with methylene blue as an adjunct to mechanical debridement [38]. In addition, a split-mouth clinical trial investigated PBMT following orthodontic bracket removal, reporting improved short-term gingival healing (Stein et al., 2018) [39]. The wavelength of the lasers used ranged from 635 nm to 660 nm, with diode lasers employed in all cases. Treatment duration varied: some studies applied LLLT for 120 s per session, over 5 consecutive days [37], while others applied it immediately post-therapy in a single session [39,40]. Dosimetry values were variably reported; however, power output ranged from 25 to 150 mW, remaining within the low-level therapeutic window.

The clinical parameters most frequently assessed were plaque index (PI), bleeding index (BI), and Community Periodontal Index of Treatment Needs (CPITN). One study also included cytomorphometric analysis of gingival epithelial cells [40] while another investigated oral yeast presence in biofilms [38]. In addition, bleeding on probing (BOP) and the papilla bleeding index (PBI) were used to evaluate gingival inflammation in a split-mouth orthodontic study [39]. Follow-up periods ranged from 5 days to 6 months.

Methodological Quality of the Included Clinical StudiesRisk of bias was identified across all included studies (Table 3). Two studies [38,39] received a moderate overall risk-of-bias rating. Both carried out randomization, with one [39] additionally adopting a patient-blinded split-mouth design and explicitly confirming the absence of adverse effects. The remaining two studies [37,40] were rated as high-risk, as neither randomization nor blinding procedures were described. Across all four studies, the reporting of dropouts and adverse effects was either absent or insufficiently detailed.

## 4. Discussion

This narrative review summarizes current evidence on low-level laser therapy for gingival inflammation across experimental, preclinical, and pediatric clinical research.

It characterizes the breadth and nature of research, revealing a progressive translational pathway from cellular mechanisms to early clinical application, while highlighting substantial gaps in standardization and pediatric-specific investigation.

**Figure 2:**
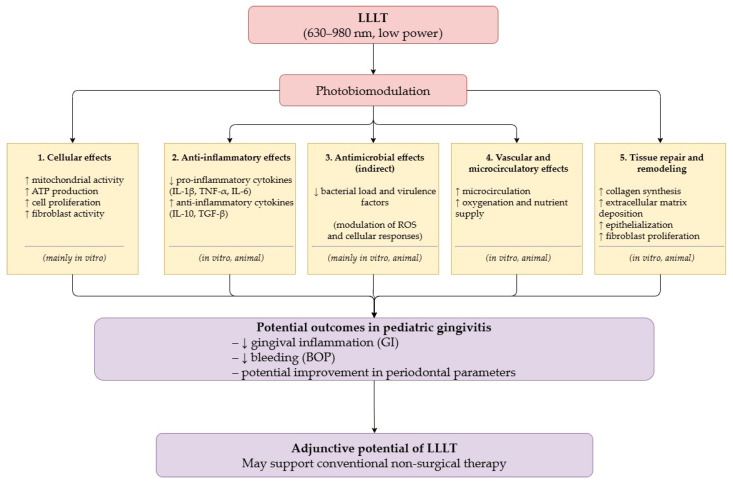
Proposed mechanisms of action of LLLT in pediatric gingival inflammation.

The in vitro studies provide foundational insights into cellular mechanisms. Human gingival fibroblasts exposed to different wavelengths and energy densities demonstrated enhanced proliferation, improved viability, and reduced pro-inflammatory cytokines (IL-1β, IL-6, TNF-α) [18,19,20,21,22,23,25,26]. Antimicrobial studies showed parameter-dependent reductions in *S. mutans* and *C. albicans* biofilms [28]. However, findings regarding antimicrobial efficacy remain inconsistent across studies, likely due to variability in laser parameters, microbial composition, experimental conditions, and the predominance of short-term mono-culture experimental models. The proposed biological mechanisms and potential clinical effects of LLLT in pediatric gingival inflammation are summarized schematically in Figure 2. The arrows represent the proposed sequence of biological and clinical effects linking LLLT-induced photobiomodulation to potential outcomes in pediatric gingivitis.

Although several mechanisms have been proposed, including modulation of inflammatory cytokines, mitochondrial activity, oxidative stress, and cellular proliferation, the relative contribution of each pathway in pediatric gingival tissues remains uncertain.

Animal models bridged cellular findings to tissue-level outcomes. Despite promising results, critical limitations include: the acute nature of ligature-induced inflammation versus chronic pediatric gingivitis, absence of developing dentition models, and short follow-up periods (maximum 30 days) insufficient to assess long-term stability. An important limitation of the animal studies literature is that most of the studies employed periodontitis models (primarily ligature-induced) [30,31,34,35,36] rather than gingivitis-only models [32,33]. While these models assess gingival inflammation as a component, they involve bone loss and attachment loss not typically present in pediatric gingivitis. Therefore, extrapolation of findings from periodontitis models to pediatric gingivitis should be interpreted cautiously.

The smaller number of gingivitis-only animal studies is mainly related to methodological constraints. Mild gingival inflammation in animals tends to resolve quickly and is difficult to standardize, whereas ligature-induced periodontitis produces a more stable and reproducible inflammatory model. Consequently, most preclinical research has focused on periodontitis models, despite their limited direct applicability to reversible pediatric gingival inflammation.

The four pediatric clinical studies (ages 7–19, *n* = 13–130 per study) provide preliminary evidence of feasibility and short-term efficacy, reporting improvements in PI, BI, and CPITN when LLLT or aPDT was used adjunctively. However, substantial methodological heterogeneity limits interpretation: treatment protocols varied from single-session to 5-day applications, wavelengths ranged from 635 to 660 nm and power outputs from 25 to 150 mW, and energy densities were inconsistently reported. Follow-up periods ranged from 5 days to 6 months, with most studies focusing on short-term outcomes, leaving questions about sustained benefits unanswered [37,38,39,40]. Interpreting the outcomes of the included studies requires accounting for several constraints. Studies [38,39] employed more controlled designs, yet small sample sizes and brief follow-up periods limit the extent to which their findings can be generalized to long-term clinical practice. In two studies [37,40], the lack of randomization and blinding makes it difficult to exclude confounding as a contributing factor to the reported results, a limitation that is not always sufficiently acknowledged in this type of research. The short observation windows common to both further narrow the clinical relevance of their conclusions. Perhaps most consistently problematic across all four studies is the inadequate documentation of dropouts and adverse effects, which leaves both attrition bias and patient safety difficult to evaluate properly.

Overall, the findings align with the adult periodontal literature showing short-term clinical benefits from adjunctive LLLT, though long-term efficacy remains uncertain. Studies in adults with type 2 diabetes demonstrated improvements in both periodontal parameters and glycemic control (HbA1c), relevant for pediatric practice given that children with diabetes may present with increased gingival inflammation [41]. However, these findings should be considered indirect evidence when extrapolated to pediatric populations, given the biological and clinical differences between adult periodontitis and pediatric gingival inflammation. The orthodontic applications identified in this review contribute to the growing literature showing LLLT and aPDT as promising adjuncts for managing appliance-induced inflammation in adolescents [38,39,42]. However, a randomized split-mouth clinical trial in adults reported that adjunctive LLLT during non-surgical periodontal therapy did not provide additional clinical benefits over SRP alone within a 12-week follow-up period, despite significant improvements over time in both groups [24].

Compared to other adjunctive therapies (chlorhexidine rinses, herbal preparations [13], probiotics [14]), LLLT offers distinctive advantages: a non-pharmacological approach avoiding antimicrobial resistance concerns, being essentially non-invasive with high pediatric acceptability, and immediate in-office application eliminating home-based adherence challenges [37]. However, LLLT has several disadvantages: higher equipment costs, requiring provider training, and lack of standardized pediatric protocols. Currently, no specific AAPD or EAPD guidelines recommend the routine use of LLLT for the management of pediatric gingivitis, further highlighting the need for standardized clinical protocols and stronger pediatric evidence.

Critical evidence gaps limiting clinical translation were identified. Only four clinical studies, comprising a total of 279 pediatric participants, are currently available, which limits the robustness of effect estimates [37,38,40]. No pediatric dose-finding studies exist; parameter selection appears based on the adult literature rather than pediatric optimization. Most studies assessed outcomes within days to weeks [37,40], with only one extending to 6 months [38], inadequate for conditions requiring long-term management. Studies included wide age ranges (7–19 years) without examining whether responses differ by developmental stage, hormonal status, or dentition phase. Despite evidence from adult studies suggesting potential benefits in medically compromised patients, pediatric research on gingivitis is limited, and, to the best of our knowledge, studies examining children with diabetes, immunocompromised status, or other systemic conditions remain scarce. Complete parameter reporting was inconsistent, with many studies omitting essential dosimetric information (beam diameter, spot size, irradiation mode) [37,38,40].

The currently available pediatric clinical evidence should be interpreted cautiously. Although current findings suggest potential short-term benefits of adjunctive LLLT in reducing gingival inflammation, the available evidence remains insufficient to support definitive clinical recommendations or standardized pediatric treatment protocols.

## 5. Conclusions

LLLT appears to be a safe and well-tolerated adjunctive approach for the management of gingival inflammation in children and adolescents, with evidence suggesting improvements in clinical inflammatory parameters when combined with conventional therapy. Its non-invasive nature and high acceptability make it particularly suitable for pediatric patients.

LLLT should not replace standard preventive and therapeutic measures, but may enhance treatment outcomes, especially in cases where plaque control is challenging. However, the currently available pediatric evidence remains limited by the small number of clinical studies, heterogeneous treatment protocols, inconsistent dosimetric reporting, lack of pediatric dose-finding studies, and predominantly short-term follow-up periods. Further well-designed pediatric clinical trials with standardized laser parameters and long-term outcome assessment are required before routine clinical implementation can be recommended.

## Figures and Tables

**Table 1:** Assessment of low-level laser therapy efficacy in in vitro models.

Study	Design	Samples	Laser Parameters	Irradiation Protocol	Results	Observations
Tanum et al. (2024) [27]	In vitro experimental study	Human gingival keratinocytes (HGKs) challenged with viable oral microbes (*S. oralis*, *S. aureus*, *C. albicans*), single- and multispecies models	Source: LED-based photobiomodulation Wavelengths: 615 nm (red) and 880 nm (near-infrared)	Mode: continuous 90-min pre-irradiation before microbial challenge	PBMT improved HGK viability under microbial challenge. PBMT reduced inflammatory signaling and intracellular ROS. Both doses enhance barrier function of microbe-challenge HGKs.	PBMT exerted combined anti-inflammatory and antimicrobial effects. Near-infrared light showed greater efficacy than red light.
Alhazmi et al. (2023) [26]	In vitro experimental study	Human gingival-derived mesenchymal stem cells (GMSCs)	Source: GaAlAs diode laser Wavelength: 980 nm Fluence: 1.5, 3 J/cm^2^	Mode: continuous Analysis at 24, 48, and 72 h	1.5 J/cm^2^ led to higher cell viability than 3 J/cm^2^. Both laser doses were non-toxic. Both doses enhanced osteogenic and odontogenic differentiation.	Low-dose photobiomodulation (1.5 J/cm^2^) is more cytocompatible than higher dose. LLLT promotes osteogenic and odontogenic potential of GMSCs.
Papadelli et al. (2021) [23]	In vitro experimental study	Control group Primary human gingival fibroblasts (hGFs) challenged with P. gingivalis lipopolysaccharide (LPS) hGFs with LPS + diode laser hGFs with LPS + Nd: YAG laser	Source: diode laser Wavelength: 810 nm Source: Nd:YAG laser Wavelength: 1064 nm	Mode: continuous Applied after 24 h LPS challenge Single irradiation session Analysis at 24, 48, and 72 h	Both wavelengths induced proliferation in naïve cells (*p* < 0.05). Both lasers decreased IL-6 & IL-8 levels particularly within the 48 h.	Both 810 nm and 1064 nm wavelengths exhibit anti-inflammatory effects of gingival fibroblasts.
Ladiz et al. (2020) [21]	In vitro experimental study	Human gingival fibroblasts (Pasteur Inst. Cell Bank, Iran)	Source: diode lasers Wavelength: 810 nm, 940 nm, combined 810 + 940 nm Fluence: 0.5, 1.5, 2.5 J/cm^2^ Power output: 100 mW	Mode: continuous for 2.5, 7.5, and 12.5 s according to fluence Single irradiation session Analysis at 24, 48, and 72 h	810 nm, 0.5 J/cm^2^: increased cell viability at 24 h. 940 nm, 0.5 J/cm^2^: suppressed cell viability. No single wavelength boost at 1.5 or 2.5 J/cm^2^. Combined wavelengths: significant increased proliferation at 1.5 & 2.5 J/cm^2^ vs. control at 48 & 72 h.	Combined wavelengths yielded superior proliferative effect. Single wavelengths had varied/short-term impact.
Lee et al. (2019) [22]	In vitro experimental study	Human gingival fibroblasts cultured in high-glucose medium (35 mM)	Source: diode laser; Low-level laser irradiation Wavelength: 660 nm	Mode: continuous Single irradiation session Analysis at 24 h	LLLT reduced expression of TNF-α, IL-1β, IL-6, and IL-8 in the hyperglycemic hGFs.	LLLT has potential to reduce hyperglycemia-induced inflammation in gingival fibroblasts.
Harorli et al. (2019) [20]	In vitro experimental study	Primary human gingival fibroblasts ± 1 µg/mL LPS	Source: diode laser Wavelength: 940 nm Fluence: 0.84, 1.4, 1.97 J/cm^2^	Mode: continuous For 20 s 3 times application at 24 h intervals Analysis at 24, 48, and 72 h	High dose (1.97 J/cm^2^) suppressed IL-6, IL-8. Lower doses (0.84, 1.4 J/cm^2^) Increased cytokine levels.	Demonstrates dose-dependent anti-inflammatory effect under inflammatory conditions.
Lee et al. (2017) [25]	In vitro experimental study	Human periodontal ligament cells stimulated with LPS from *Porphyromonas gingivalis* or *E. coli*	Source: GaAlAs laser Wavelength: 660 nm Fluence: 8 J/cm^2^	Mode: continuous For 528 s Single irradiation session Irradiation immediately after LPS stimulation	Decreased pro-inflammatory cytokines (TNF-α, IL-1β, IL-6, IL-8). Increased cAMP levels.	LLLT reduced inflammation via cAMP/NF-κB signaling pathway in human periodontal ligament cells.
Fronzafar et al. (2013) [19]	In vitro experimental study	Human gingival fibroblasts cultured in 96-well plates	Source: GaAlAs diode laser Wavelength: 810 nm Fluence: 4 J/cm^2^ Power output: 50 mW	Mode: continuous for 32 s Applied after 24 h incubation 3 consecutive days Analysis at 24, 48, and 72 h	Day 1: No significant difference in cell proliferation compared to control group. Days 2 & 3: Significant increase in cell proliferation in irradiated group. Significant increase in collagen type I gene expression on Day 3.	Laser therapy stimulated both cell proliferation and collagen type I gene expression.
Basso et al. (2012) [18]	In vitro experimental study	Cultured human gingival fibroblasts	Source: diode laser Wavelength: 780 nm Fluence: 0.5, 1.5, 3, 5, 7 J/cm^2^ Power output: 40 mW	Mode: continuous every 24 h 3 applications Analysis after 24 h	Increased cell metabolism and proliferation at 0.5 & 3 J/cm^2^. Higher doses (5 & 7 J/cm^2^) showed no added benefit.	LLLT promoted fibroblast proliferation and migration at optimal doses at 0.5 and 3 J/cm^2^.
Basso et al. (2011) [28]	In vitro experimental study	*S. mutans* and *C. albicans* biofilms	Source: InGaAsP diode laser Wavelength: 780 nm Fluence: 5, 10, 20 J/cm^2^	Mode: continuous 250, 500, and 1000 s according to fluence Single irradiation session Analysis after 15 h	Decreased viability and growth of *S. mutans* and *C. albicans* biofilms. *S. mutans* resisted in dual-species setting. Decreased hyphal formation in *C. albicans.*	LLLT shows antimicrobial potential, but species interactions affect efficacy.

LLLT—low level laser therapy; IL-6—interleukin-6; IL-8—interleukin-8; HGKs—human gingival keratinocytes; PBMT—photobiomodulation therapy; ROS—reactive oxygen species; GMSCs—gingival-derived mesenchymal stem cells; GaAIAs—gallium–aluminum–arsenide; hGFs—human gingival fibroblasts; LPS—lipopolysaccharide; Nd:YAG—Neodymium-doped Yttrium–Aluminum–Garnet; TNF-α—tumor necrosis factor-alpha; IL-1β—interleukin-1beta; cAMP—cyclic adenosine monophosphate; NF-κB—nuclear factor kappa B; InGaAsP—indium gallium arsenide phosphide.

**Table 3:** Evaluation of the effectiveness of low-level laser therapy in pediatric clinical studies regarding gingivitis reduction.

Study	Design	Samples	Laser Parameters	Irradiation Protocol	Results	Observations	Methodological Quality Assessment
Malik & Alkadhi (2020) * [38]	Randomized, controlled pediatric clinical trial	36 adolescents (mean age 16.7) Control group (*n* = 18): Mechanical debridement (MD) alone Test group (*n* = 18): MD + aPDT	Source: diode laser Wavelength: 660 nm Fluence: 0.0125 J/cm^2^ Power output: 150 mW Cumulative dose: 3 J Exposure duration: 60 s	MD via ultrasonic scaling aPDT: methylene blue (400 μg/mL) photosensitizer, 15 s waiting + visible light irradiation (diode laser)	Both groups showed comparable reductions in gingival index after 6 months. Only the test group had a significant decrease in salivary yeast counts (CFU/mL) from baseline to 6 months (*p* < 0.05).	aPDT as an adjunct to mechanical debridement effectively reduced oral yeast colonization. aPDT did not provide additional benefits in reducing gingival inflammation compared to MD alone.	small sample size; performed randomization; blinding procedures—not clearly specified; 6-month follow-up; no dropouts reported; adverse effects were not explicitly or systematically reported.
Igic et al. (2020) [37]	Controlled, pediatric clinical study	100 children with permanent dentition (aged 13–17 years) with catarrhal gingivitis Group I (HA- hyaluronic acid): *n* = 50 Group II (LLLT): *n* = 50	Source: diode laser Wavelength: 635 nm Power output: 25 mW Exposure duration: 120 s	Basic therapy: oral hygiene + plaque/tartar removal Group I: basic therapy + topical hyaluronic acid gel daily for 7 days Group II: basic therapy + LLLT, 5 consecutive days	Both groups showed significant reductions (*p* < 0.001) in plaque index, bleeding index, and CPITN after treatment. Post-treatment CPITN was significantly lower in the LLLT group vs the HA group (*p* < 0.05).	Both adjunctive therapies (LLLT & HA) significantly enhanced outcomes versus basic care alone. LLLT provided a modest but statistically significant advantage over HA in improving treatment needs. No adverse effects were reported.	no randomization; no blinding procedures reported; no dropouts reported; short-term assessment period; adverse effects were not explicitly or systematically reported.
Stein et al. (2018) [39]	Randomized, patient-blinded, split-mouth, controlled pediatric clinical trial	13 adolescents aged 12–19 (mean age 16.15 years) after fixed orthodontic treatment with multi-bracket appliances	Source: diode laser Wavelength: 660 nm Fluence: 52 J/cm^2^ Power output: 100 mW Power density: 100 mW/cm^2^ Energy density: 2 J/cm^2^ per point Exposure duration: 20 s per point	Bracket debonding + professional tooth cleaning in both sides Laser side: one maxillary quadrant treated with LLLT Control side: the other upper quadrant with sham laser application	Both laser and control sides showed significant reductions in BOP and PBI from baseline to follow-up (*p* < 0.05). At 5 days post-treatment, BOP and PBI values were significantly lower on the PBMT side compared with the control side (*p* < 0.05).	PBMT accelerated gingival healing following orthodontic appliance removal. No adverse effects or patient discomfort were reported. PBMT provided additional short-term clinical benefits beyond mechanical plaque removal alone.	small sample size; performed randomization; patient-blinded, split-mouth design; no dropouts reported; short-term follow-up period (5 days); no adverse effects or patient discomfort observed.
Igic et al. (2012) [40]	Controlled, pediatric clinical study	130 children (7–14 years) Group 1 (with chronic catarrhal gingivitis): basic therapy (*n* = 50) Group 2 (with chronic catarrhal gingivitis): basic therapy + LLLT (*n* = 50) Group 3 (control): healthy gingiva (*n* = 30)	Source: diode laser Wavelength: 635 nm Power output: 25 mW Exposure duration: 120 s Power density: 200 mW/cm^2^	Basic therapy: oral hygiene + plaque removal LLLT group: adjunct laser treatment immediately post-therapy	Cytomorphometry: Significant reduction in the size of the nuclei of stratified squamous epithelial cells of the gingiva (*p* < 0.001) in both treatment groups. Only the LLLT group restored nuclear size to match healthy controls. Clinical indices: Gingival status improved after hygiene therapy alone; LLLT accelerated normalization to healthy reference levels.	LLLT provided additional normalization of epithelial cell morphology beyond basic therapy. Clinical improvement was more pronounced and faster in the LLLT group.	no randomization; no blinding procedures reported; no dropouts reported; short-term assessment period; adverse effects were not explicitly or systematically reported.

MD—mechanical debridement; aPDT—antimicrobial photodynamic therapy; CFU/mL—colony-forming units per milliliter; HA—hyaluronic acid; LLLT—low-level laser therapy; BOP—bleeding on probing; PBI—papilla bleeding index; PBMT—photobiomodulation therapy; CPITN—Community Periodontal Index of Treatment Needs. * Malik & Alkadhi (2020) evaluated antimicrobial photodynamic therapy (aPDT) combined with mechanical debridement rather than isolated LLLT. However, because aPDT also involves the application of low-intensity light/laser energy as an adjunctive photobiomodulatory approach, the study was considered relevant for the overall discussion of light-based adjunctive therapies in pediatric gingival inflammation.

## Data Availability

No new data were created or analyzed in this study. Data sharing is not applicable to this article.

## Abbreviations

The following abbreviations are used in this manuscript:

LLLT	Low-Level Laser Therapy
PBMT	Photobiomodulation Therapy
SRP	Scaling and Root Planing
hGFs	Human Gingival Fibroblasts
aPDT	Antimicrobial Photodynamic Therapy
BOP	Bleeding on Probing
PBI	Papilla Bleeding Index
BI	Bleeding Index
PI	Plaque Index
GI	Gingival Index
CPITN	Community Periodontal Index of Treatment Needs
TNF-α	Tumor Necrosis Factor-alpha
IL-6	Interleukin-6
IL-8	Interleukin-8
IL-1β	Interleukin-1 beta
